# Emergency Department Changes to Combat COVID-19 in Oman

**DOI:** 10.1017/dmp.2021.38

**Published:** 2021-02-16

**Authors:** Muhammad Faisal Khilji, Mehmood Al Jufaili

**Affiliations:** 1 Emergency Department, Royal Lancaster Infirmary, University Hospitals of Morecambe Bay NHS Foundation Trust, Lancaster, United Kingdom; 2 Department of Emergency Medicine, Sultan Qaboos University Hospital, Muscat, Sultanate of Oman

**Keywords:** COVID-19, emergency, Oman, pandemics, personal protective equipment

## Abstract

Our hospital is one of the tertiary care hospitals in Oman receiving coronavirus disease (COVID-19; C19) patients. To meet the expected surge of patients, a number of changes was made to the emergency department (ED), especially regarding capacity building and patient flow. At first, few changes were made to the main ED, which mainly includes the addition of a COVID suspect room with the use of a separate resuscitation area. The major drawback of the abovementioned system was the inability to see more than 2 patients simultaneously. A later separate COVID emergency department (CED) was used. In the CED, pending admissions was the major problem, as the C19 ward and C19 intensive care unit were becoming full; this problem was solved through central command help. In the normal ED, the main problem was the presentation of C19-positive patients sometimes hiding their symptoms and reaching inside the main ED, exposing the staff and patients. In order to combat this problem, all patients with an acute respiratory problem, even if C19 is not suspected, were taken to the corner cubicle. In this report, the changes made in the ED to combat C19 spread are discussed.

## Introduction

Severe acute respiratory syndrome coronavirus 2 (SARS-CoV-2) is responsible for the 2019 coronavirus disease (COVID-19; C19). It was initially reported in December 2019 in Wuhan, Hubei Province, China, where a cluster of pneumonia cases was reported without any known cause.^1,2^ On January 2020, China declared a novel coronavirus as a cause of these pneumonia cases that continued to increase exponentially and soon spilled all over the world.^1^ On January 30, 2020, the World Health Organization (WHO) declared a Public Health Emergency of International Concern and, on March 11, 2020, the WHO declared C19 a pandemic. The first case in Oman was reported among 2 females returning from Iran.^3^ The Government of Oman responded by setting up an incident command chain and hotline numbers for the general public. The 2 main tertiary care hospitals in Oman, The Royal Hospital and Sultan Qaboos University Hospital, stopped routine outpatient clinics and surgeries on March 16, 2020.

## Floor Plan Changes and Patient Flow

### Initial Phase – Alterations in the Main Emergency Department (March to April)

**Figure 1.**
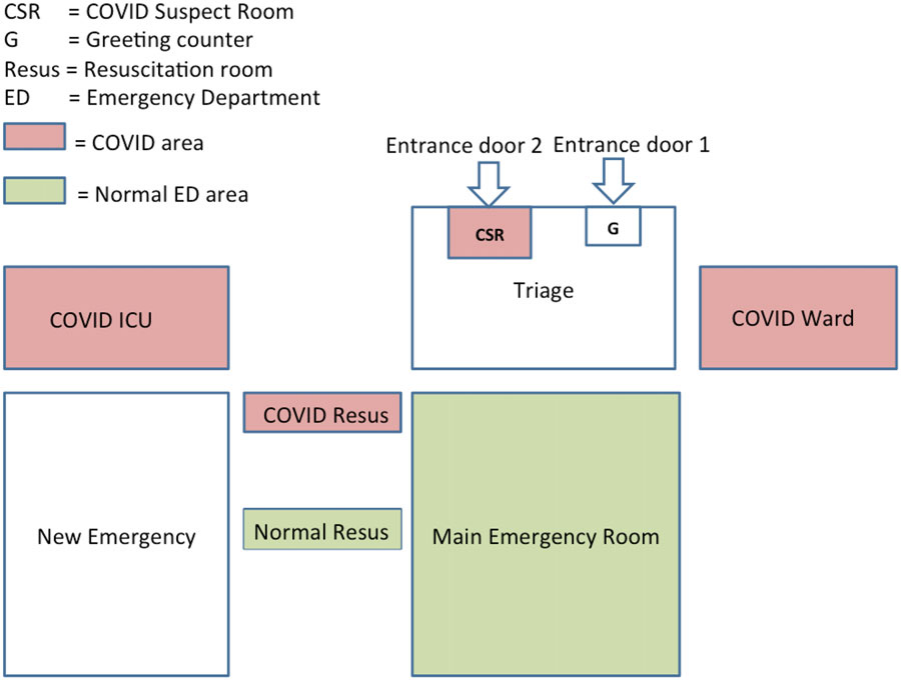
ED floor plan changes for the initial phase of the C19 setup.

Our hospital covers the northern districts of Muscat Governorate where the number of cases is comparatively less compared with the southern districts. A number of steps was taken to cope with the possible surge of C19 patients. A plan was formulated regarding capacity building, patient flow, and creation of beds for C19 patients, and establishing necessary training for the staff. Extra staff was allocated to the emergency department (ED) by the hospital administration upon ED request, and extra personal protective equipment (PPE) was indented from the hospital stores with a revision of PPE supply demand for the next 3 months. A front desk was added to the main ED door, along with the addition of an extra COVID suspect room (CSR) dedicated for suspected C19 patients, to the next ED entrance door (Figure 1).

**Figure 2.**
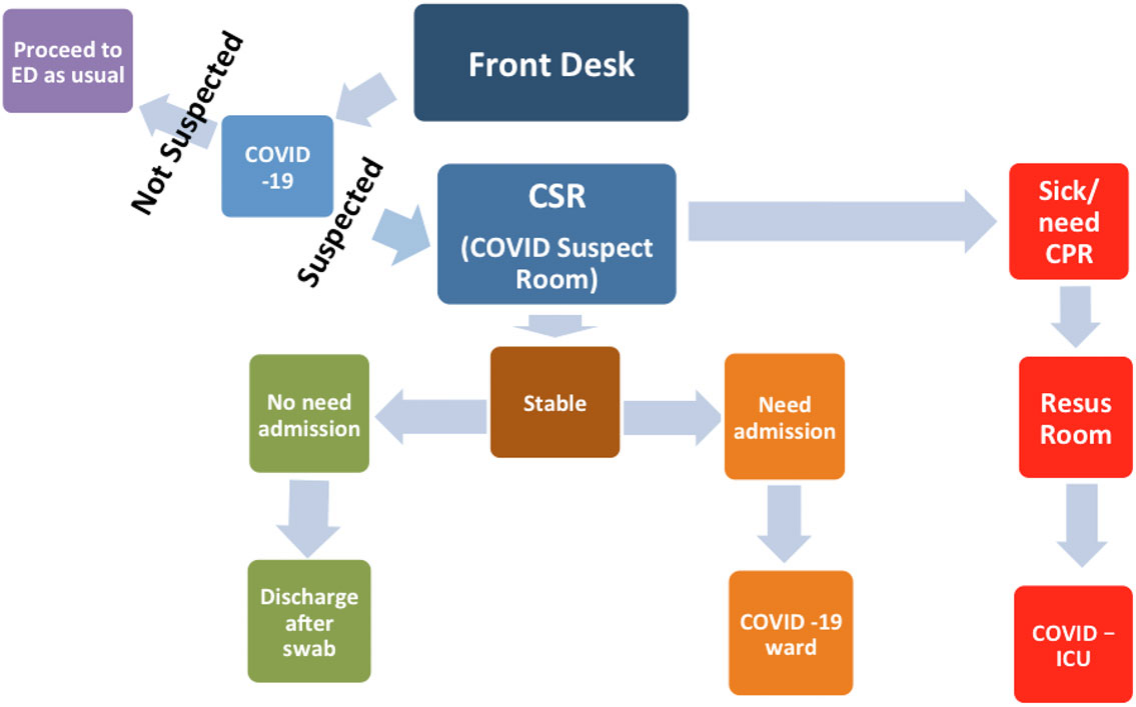
C19 patients flow in the initial phase.

A separate resuscitation area, other than the resuscitation area of the normal ED, is allocated in the ED for C19 patients. A C19 ward and a C19 intensive care unit (ICU), each having 10 beds, were established in the hospital. In terms of flow, a greeting nurse takes a quick history assessing whether the patient is a C19 suspect. If suspected of C19, the patient is moved directly to the CSR in the second entrance door where a dedicated C19 doctor assesses the patient (see Figure 1). If stable enough to go home, the patient is discharged after taking a C19 swab, with instruction to quarantine for 2 weeks if the test is reported positive, which will be communicated to the patient the next day. If the C19 doctor thinks that the patient needs admission, then the doctor can admit the patient directly to the C19 ward. If the patient is unstable, then the patient is taken to the C19 resuscitation area in the ED, and after stabilization the patient is admitted to the C19 (or COVID-19) ICU (Figure 2).

In the latter 2 cases, the C19 swab will be taken after admission as both C19 ward and C19 ICU have negative pressure ventilation. All C19-related ED areas were covered by separate C19 nurses (4 to 5) in each shift. Emergency beds can be increased up to 52 beds from the present number of 24 beds; as the new ED building is ready but not operational, we could use 28 extra beds of the new ED building (see Figure 1). The changes made to the ED helped relieve the possible surge effect of C19 cases and helped keep C19 and non-COVID emergency department (NCED) patients separate to prevent the spread of C19 in the hospital. However, since the beginning of the C19 crisis, the total number of patients visiting the ED has dropped. As new patients are not being admitted due to the suspension of routine surgeries and routine clinics, the number of admission beds has increased.

### Later Phase – COVID ED Setup (May to August)

**Figure 3.**
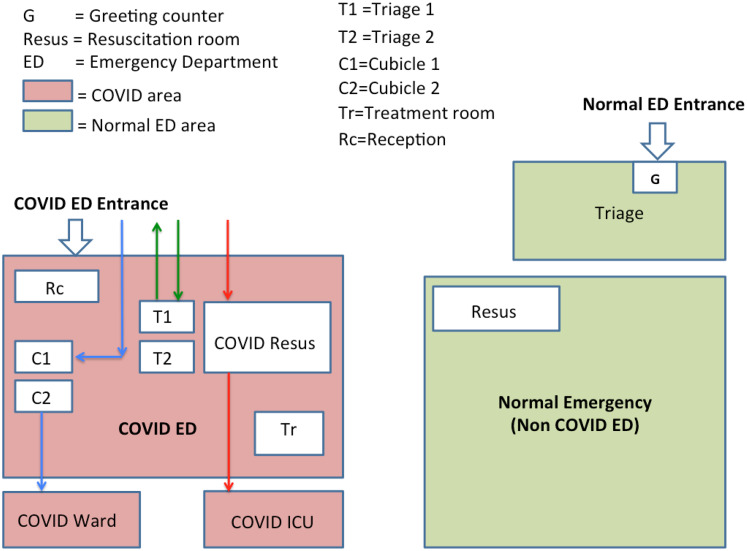
CED floor plan (diagrammatic presentation): dischargeable patient flow (green arrows), C19 ward admission patient flow (blue arrows), C19 ICU admission patient flow (red arrows).

This system worked well, initially, when the number of patients was less, about 5 to 10 patients per day; by the end of May, the number of patients started increasing. The major drawback was the inability to see more than 2 patients simultaneously due to the limited space in the CSR and extra patients had to wait in their cars. In order to cope with this situation, the new ED, as mentioned earlier, is used from the last week of May to August and labeled as the COVID emergency department (CED). The old ED was working as the “normal” or NCED, catering to all non-COVID emergencies. A C19 signboard is installed outside the main entrance of the new CED, directing all C19 patients there. However, a greeting nurse is still present on the entrance of the NCED, directing any C19-suspected patient to the CED, which has 2 triage rooms, 2 short-stay rooms, 1 treatment room, and 1 resuscitation room, each having 1 bed. Stable patients are discharged after taking a C19 swab, with instruction to quarantine for 2 weeks if the test is positive, which is communicated to the patient the next day. Sick patients requiring admission are kept in a short-stay cubicle or treatment room if any procedure is required and the patient is admitted to the C19 ward. The very sick patients are kept in the resuscitation area and later admitted to the C19 ICU. A C19 GeneXpert test is done for all patients requiring possible admission, which is reported by the on-call virologist within 2 hours. C19-positive patients are admitted to the C19 ward or C19 ICU accordingly; however, if the C19 test is negative for a patient requiring admission, then the patient is shifted to the NCED and admitted from there as per protocol (Figure 3).

**Figure 4.**
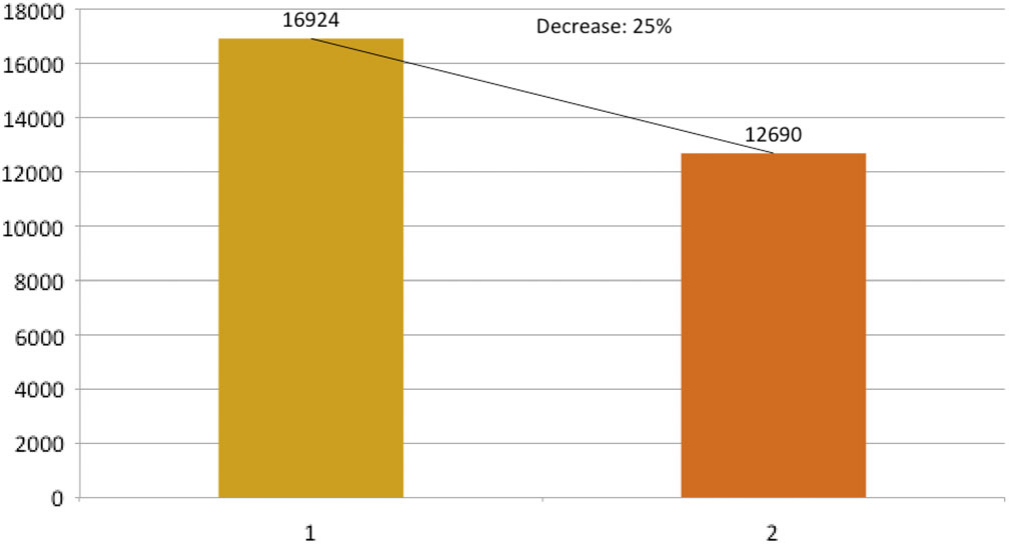
Decrease in the number of patients during March to June 2020. Bar 1 shows 2019 and bar 2 shows 2020 total number of patients from March to June.

**Figure 5.**
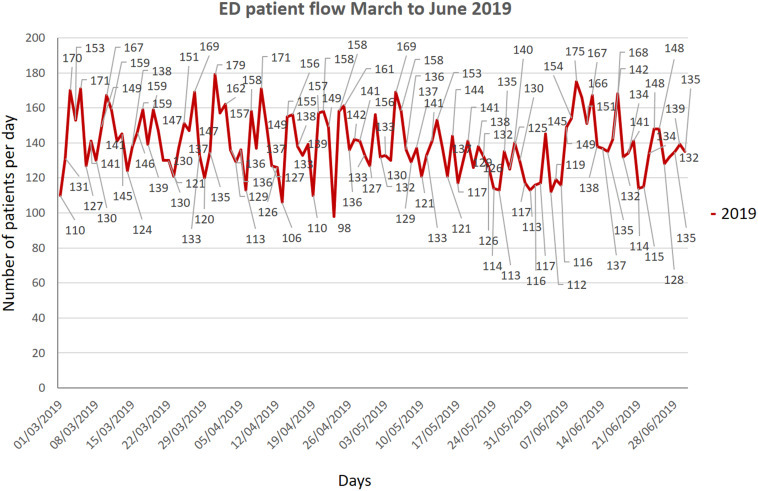
Total number of patients from March to June 2019.

**Figure 6.**
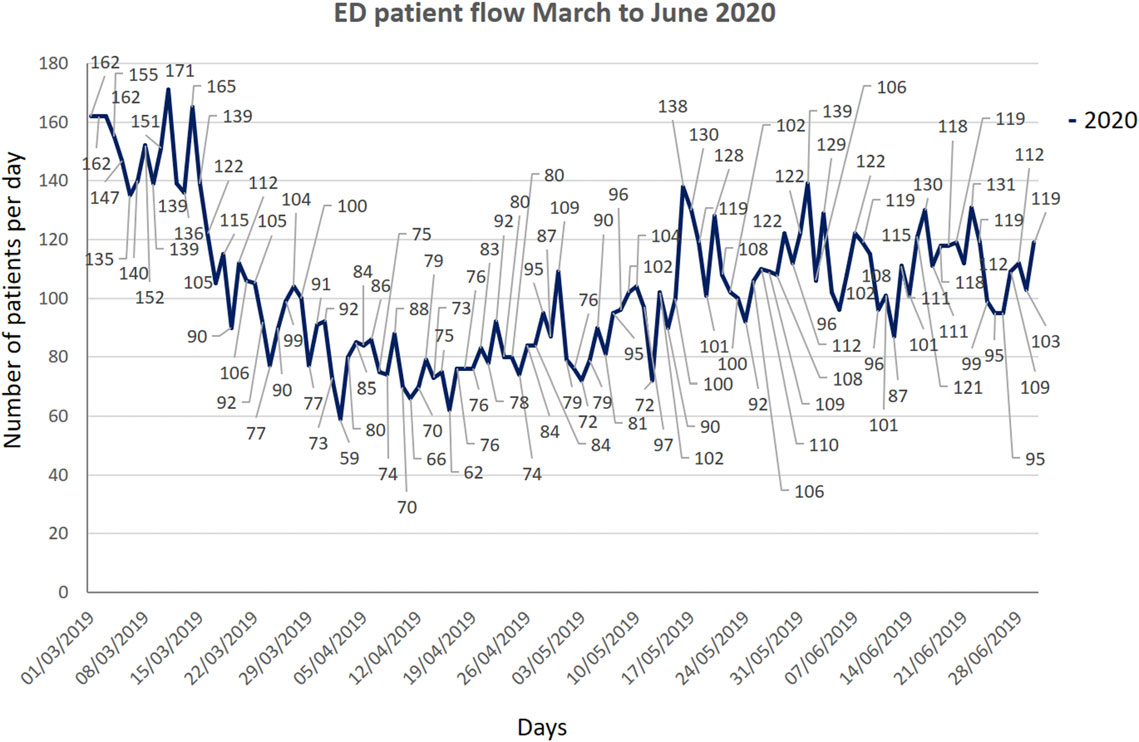
Total number of patients from March to June 2020.

**Figure 7.**
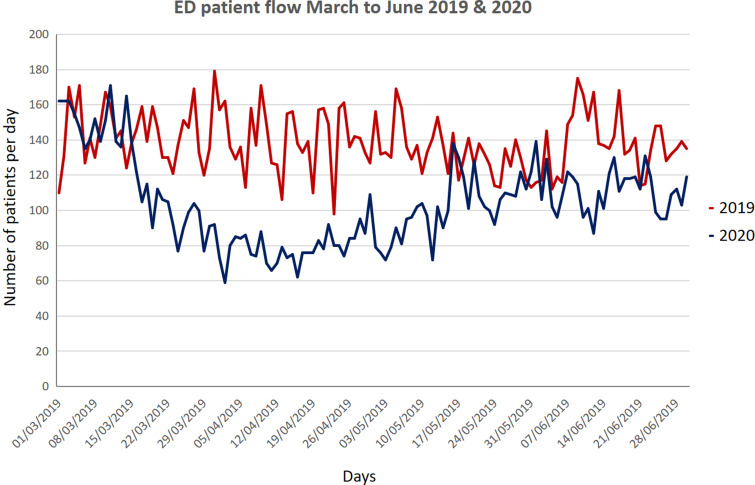
Patient flow comparison of March to June 2019 and 2020.

It is worth noting here that more than 70% of the patients presenting to the CED require admission as only sick patients come to the hospital and patients with trivial symptoms report to the local health centers. Every room of the CED is fumigated by housekeeping staff wearing full PPE, and closed for 1 hour, once C19-positive patient leaves the room. Overall, the total number of patients, when compared with the same period of time in 2019, fell by about 25% (Figure 4). The total number of patients from March to June 2020 were compared with same months of 2019, and graphs were generated in Microsoft® PowerPoint® 2016 version (16.0.4266.1001) MSO (16.0.5032.1000) 32 bit (Figures 5, 6, and 7).

## Equipment

### Personal Protective Equipment

PPE is the specialized equipment or clothing worn to minimize exposure to hazards.^4^ The donning and doffing of PPE are vital for maintaining the health of health care workers and in preventing the spread of infection.^5^ The staff using PPE in exposed areas includes doctors, nurses, paramedics, emergency medical services staff, medical orderlies, housekeeping, and food serving staff.

**Figure 8.**
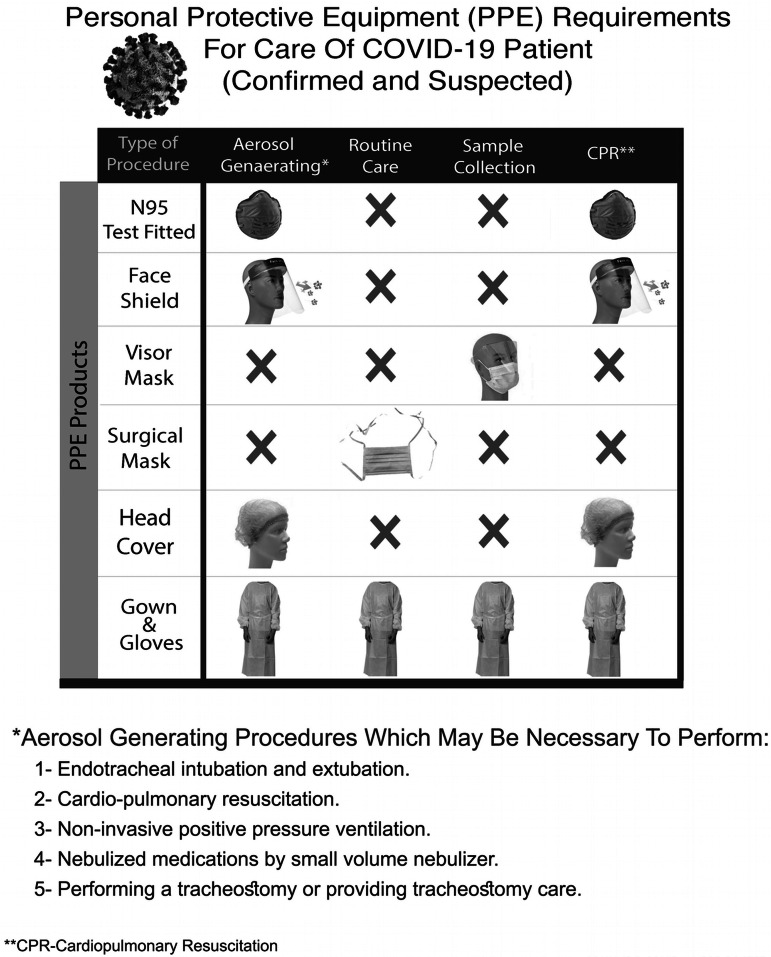
Hospital-approved PPE used in the ED.

The following PPE is used by the staff when exposed to suspected or confirmed C19 patients (Figure 8).

- Disposable gown
- Face mask N95 (fit tested)
- Face shield
- Disposable cap
- Shoe cover
- Surgical masks

Every staff is issued 1 N95 mask and 1 face shield per shift as part of PPE conservation policy of the hospital. Gown gloves and shoe covers can be changed according to the needs. The face shield should be cleaned with a wipe every time after exposure to a C19-suspected or C19-positive patient.

The donning and doffing procedures were defined and staffs were trained for that; usually, donning and doffing were performed in areas outside every CED room, in the corridor. PPE was disposed in biohazard bins; however, N95 masks were disposed in a recycle bin from where they were collected for reuse by using the hydrogen peroxide method.^6^

### Other Equipment

**Figure 9.**
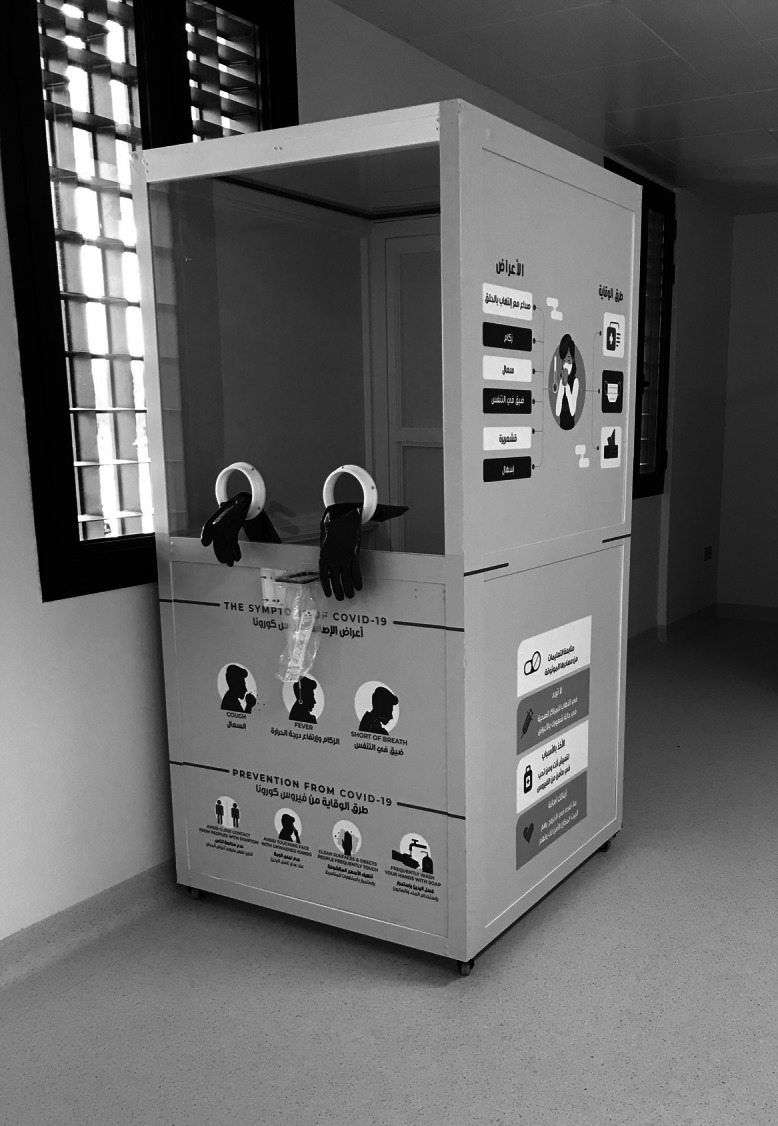
Modular booth for C-19 sampling of stable and dischargeable patients.

CED has the same equipment as NCED with the difference that C-MAC machine was used for intubation of the patient, which allows the doctor to keep distance from the patient, and the plastic sheet is used over the patient during intubation, while doctor is putting hands below the sheet for intubation. Ultrasound machine and other important equipment were also covered with a protective sheet. Food trolleys were also provided with thick plastic sheet barriers with staff handling food trolleys wearing PPE in the CED. A C19 single modular booth is also available for taking a nasopharyngeal swab of stable, suspected patients (Figure 9).

## Staffing

**Figure 10.**
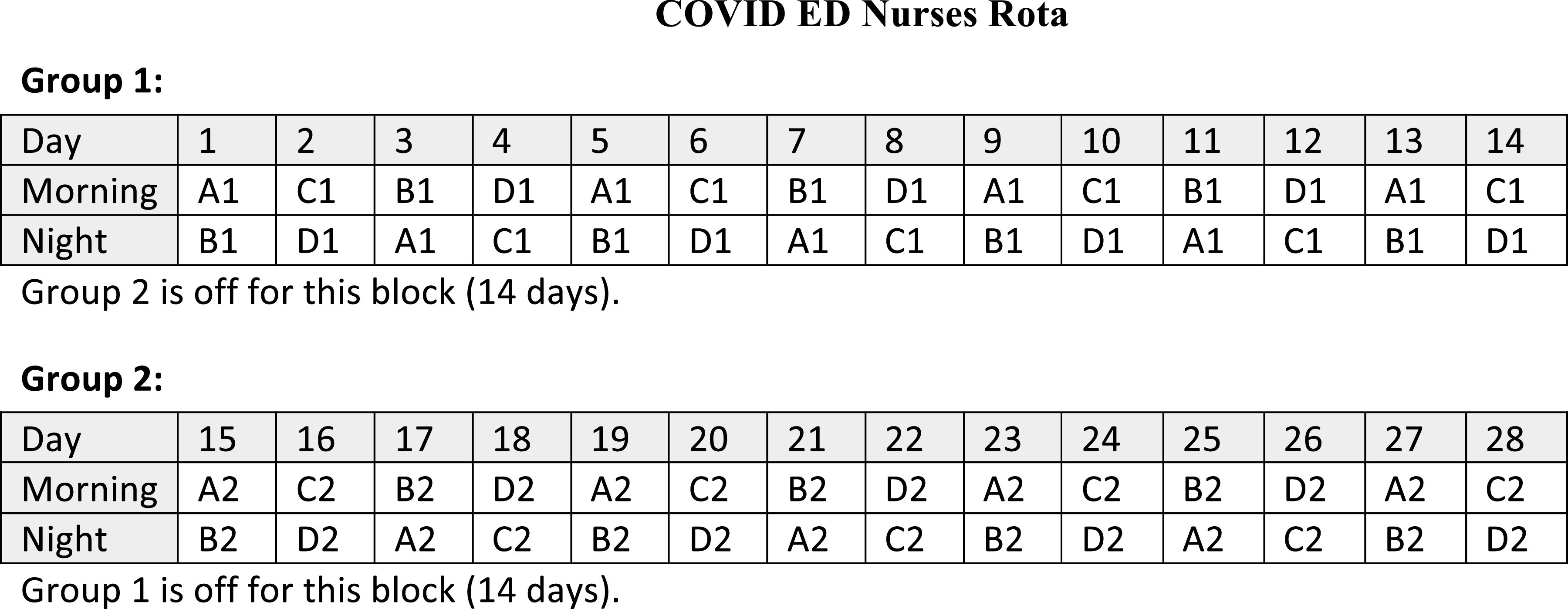
Each group (1 and 2) comprises 10 nurses with further subgroups (A and B), each having 5 nurses in each shift. Group 1 is working the first 2 weeks when group 2 is off, whereas group 2 is working the next 2 weeks when group 1 is off. Each shift (morning or night) is 12 hours.

Studies from Italy and China have shown that health care workers are extremely vulnerable to C19 infections.^7^ Prevention of C19 infection in health care staff is vital for the normal functioning of the health care system.^8^ A staff rota is an important tool to minimize the exposure of staff to the infected patients by minimizing the number of staff exposed to the infected patients at any given point.^8^ The staff rota was developed to meet these criteria, and hence 2 groups of 5 staff each were rotated alternately in a 12-hour shift for 2 weeks, then 2 weeks off and vice versa for the second group, before shifting back to the NCED (Figure 10). In every shift, 1 or 2 doctors were posted in the CED while rotating in 8-hour shifts. A swab is taken for any staff developing symptoms and treated accordingly.

## Support Services

The medical orderlies, food serving staff, and housekeeping staff posted in CED have to wear PPE while performing duties.

## Discussion

### General Discussion

C19 is a very contagious, rapidly spreading, and mostly unpredictable coronavirus-related disease.^9^ It started in Wuhan city in China in December 2019, reaching up to pandemic levels in March 2020. It is caused by the SARS-CoV-2 virus with an incubation period of 2 to 14 days, typically 5 to 6 days.^10^ C19 causes flu-like symptoms with fever, fatigue, cough, and shortness of breath, which usually recover by itself or with supportive treatment in most of the cases, but, in a few cases, it may progress to severe respiratory symptoms and multiorgan failure leading to death. Thus far, there is no specific treatment or vaccination available for C19. It spreads mainly through small droplets suspended in the air, even with normal talking by the affected person, creating a dilemma for the governments around the world as how to control its spread.^11^ PPE is a must for the medical staff due to the close proximity of medical staff to the patient, making them vulnerable to the disease. The real-time reverse transcription polymerase chain reaction (rRt- PCR) test of a nasopharyngeal swab is the main test for the diagnosis of C19.^12^ The present knowledge of epidemiology reveals that the “flattening of the epidemic curve” through mitigation and “raising the line” through greater health care capacity are the 2 main prongs of the strategy required to overcome this pandemic.^13^ Social distancing, handwashing, and wearing a mask are components of prevention for the general public, compelling governments to exercise, lockdown of cities, and closure of international borders to avoid a mass gathering as much as possible.

### Comparison With Other Studies

Our study showed a significant drop in the total number of patients, as evidenced in many other studies, like Hartnett et al., Thornton et al., and Santana et al. studies (see Figure 7).^14–16^ The main difference in our setup from other studies like Li Juan Joy Quah et al.ʼs, and Whiteside et al.’s is that, their CED and normal ED resuscitation beds are in the same physical area, whereas we separate CED with its dedicated resuscitation area; however, use of PPE is almost similar in our setup as in Quah et al.’s and Hockaday et al.’s studies.^5,17,18^ The nursing staff rota is similar in our and Quah et al.’s studies.^17^

### What Makes Our Setup Unique in the World?

Most of the hospitals have made alterations in their present floor plan setup or acquired other departments of the hospital to expand the ED, like a surgery department as in Lin Juan Joy Quah et al.’s study, whereas others have made temporary field hospital expansions to expand their ED, like Hockaday et al.’s study.^5,17^ Our setup includes a whole new ED for C19 patients in a new building, which also includes a dedicated resuscitation room (see Figure 3).

### Mistakes

The main mistake in our setup is the presence of the donning and doffing area in the corridors, as there is no separate entrance and exit for C19 staff. Ideally, donning and doffing should be at the entrance and exit of staff, respectively.^5^ We are not following any specific doffing guidelines, but other studies have shown the adherence to specific doffing guidelines as shown in the Hockaday et al.’s study.^5,19,20^ Reuse of N95 masks with hydrogen peroxide is questioned in some studies.^21^ Many studies have used separate pathways for contaminated and non-contaminated areas, which we did not.^5,22^ We have not developed a specific protocol for visitors that are mentioned in some studies.^23^

## Conclusion

In order to meet the expected surge of C19 patients, changes were made to the ED. In the first phase (March to April), the CSR with the use of a separate resuscitation area was added, but the major drawback of that system was the inability to see more than 2 patients simultaneously due to the limited space in the CSR. Later (May to July), a separate CED was set up. In the CED, pending admissions were the major problem; as the C19 ward and C19 ICU were becoming more occupied, the problem was solved through central command help. In the NCED, the main problem was the presentation of C19-positive patients, sometimes hiding their symptoms and history to the triage doctor, thus reaching inside the main ED and exposing the whole staff and patients inside the NCED. In order to combat this problem, all patients with a respiratory problem, even if not suspected, were taken to the corner cubicle. PPE conservation is attempted by reuse of N95 masks. The C19 situation is still not over, and hence a follow-up study is required once the present situation is over.

## References

[1] Wang C , Horby PW , Hayden FG , et al. A novel coronavirus outbreak of global health concern. Lancet. 2020;395:470–473. 10.1016/S0140-6736(20)30185-9. 31986257 PMC7135038

[2] Heymann DL , Shindo N. WHO Scientific and Technical Advisory Group for Infectious Hazards. COVID-19: what is next for public health? Lancet. 2020;395:542–545. doi: 10.1016/S0140-6736(20)30374-3. 32061313 PMC7138015

[3] The Straits Times. Coronavirus: Iraq, Oman confirm first cases, halt flights to Iran. February 24, 2020. https://www.straitstimes.com/world/middle-east/coronavirus-iraq-confirms-first-case. Accessed April 23, 2020.

[4] Occupational Safety and Health Administration. Personal protective equipment. No date. https://www.osha.gov/SLTC/personalprotectiveequipment. Accessed April 23, 2020.

[5] Hockaday S , Krause K , Sobieski C , et al. Protocols for personal protective equipment in a COVID-19 medical shelter. Disaster Med Public Health Prep. 2020;14(4):551–557. doi: 10.1017/dmp.2020.244. 32660678 PMC7450219

[6] Schwartz A , Stiegel M , Greeson N , et al. Decontamination and reuse of N95 respirators with hydrogen peroxide vapor to address worldwide personal protective equipment shortages during the SARS-CoV-2 (COVID-19) pandemic. *Appl Biosaf.* 2020;epub. 10.1177/1535676020919932 PMC9387741 36035079

[7] Remuzzi A , Remuzzi G. COVID-19 and Italy: what next? Lancet. 2020;395(10231):1225–1228. 32178769 10.1016/S0140-6736(20)30627-9 PMC7102589

[8] Kluger DM , Aizenbud Y , Jaffe A , et al. Impact of healthcare worker shift scheduling on workforce preservation during the COVID-19 pandemic. *Infect Control Hosp Epidemiol*. 2020;epub. doi: 10.1017/ice.2020.337. PMC7403749 32684183

[9] Centers for Disease Control and Prevention. Coronavirus disease 2019 (COVID-19). How COVID-19 spreads. 2020. https://www.cdc.gov/coronavirus/2019-ncov/prepare/transmission.html. Accessed April 23, 2020.

[10] Healthdirect. Australian Government, Department of Health. Healthdirect symptom checker. March 18, 2020. https://www.healthdirect.gov.au. Accessed April 19, 2020.

[11] Centers for Disease Control and Prevention (CDC). How COVID-19 spreads. April 2, 2020. https://www.cdc.gov/coronavirus/2019-ncov/transmission/index.html. Accessed April 2, 2021.

[12] Zhang C , Wu Z , Li JW , et al. The cytokine release syndrome (CRS) of severe COVID-19 and Interleukin-6 receptor (IL-6R) antagonist Tocilizumab may be the key to reduce the mortality. Int J Antimicrob Agents. 2020;55(5):105954. doi: 10.1016/j.ijantimicag.2020.105954. 32234467 PMC7118634

[13] Barclay E , Scott D , Animashaun A , et al. The US doesn’t just need to flatten the curve. It needs to “raise the line.” *Vox*. Archived from the original on April 7, 2020. 2020.

[14] Hartnett KP , Kite-Powell A , DeVeis J , et al. Impact of the COVID-19 pandemic on emergency department visits – United States, January 1, 2019–May 30, 2020. US Department of Health and Human Services/Centers for Disease Control and Prevention. MMWR Morb Mortal Wkly Rep. 2020;69(23):699–704. 32525856 10.15585/mmwr.mm6923e1 PMC7315789

[15] Thornton J. COVID-19: A&E visits in England fall by 25% in week after lockdown. *BMJ.* 2020;369:m1401. doi: 10.1136/bmj.m1401. 32253175

[16] Santana R , Sousa JS , Soares P , et al. The demand for hospital emergency services: trends during the first month of COVID-19 response. Port J Public Health. 2020;38:30–36. 10.1159/000507764 PMC7206358 40504165

[17] Quah LJJ , Tan BKK , Fua TP , et al. Reorganising the emergency department to manage the COVID-19 outbreak. Int J Emerg Med. 2020;13:32. 32552659 10.1186/s12245-020-00294-w PMC7298444

[18] Whiteside T , Kane E , Aljohani B , et al. Redesigning emergency department operations amidst a viral pandemic. Am J Emerg Med. 2020;38:1448–1453. 32336583 10.1016/j.ajem.2020.04.032 PMC7156950

[19] Emory University. COVID-19: conserving PPE. 2020. https://med.emory.edu/departments/medicine/divisions/infectiousdiseases/serious-communicable-diseases-program/covid-19resources/conserving-ppe.html. Accessed June 1, 2020.

[20] Centers for Disease Control and Prevention. Interim infection prevention and control recommendations for healthcare personnel during the coronavirus disease 2019 (COVID 19) pandemic. https://www.cdc.gov/coronavirus/2019-ncov/hcp/infection-control-recommendations.html. Accessed April 2, 2021

[21] Peltier RE , Wang J , Brian L , et al. Addressing decontaminated respirators: some methods appear to damage mask integrity and protective function. *Infect Control Hosp Epidemiol.* 2020;epub, 1–3. DOI: 10.1017/ice.2020.332. PMC7385316 32669135

[22] REBELEM Blog. COVID-19: a powerful message from Italy. March 20, 2020. https://rebelem.com/covid-19-a-powerful-message-from-italy/. Accessed March 21, 2020.

[23] American College of Emergency Physicians. National strategic plan for emergency department management of outbreaks of COVID-19. 2020. https://www.acep.org/globalassets/sites/acep/media/by-medical-focus/covid-19-national-strategic-plan_0320.pdf. Accessed March 21, 2020.

